# Editorial: Multimodal fusion technologies and applications in the context of neuroscience

**DOI:** 10.3389/fnins.2023.1213207

**Published:** 2023-05-16

**Authors:** Xiaomao Fan, Meiyu Qiu, Weidong Gao, Wenjun Ma, Yunpeng Cai, Hui Zhou, Dingguo Zhang, Raffaele Gravina, Jian Huang

**Affiliations:** ^1^College of Big Data and Internet, Shenzhen Technology University, Shenzhen, China; ^2^School of Information and Communication Engineering, Beijing University of Posts and Telecommunications, Beijing, China; ^3^School of Computer Science, South China Normal University, Guangzhou, China; ^4^Research Center for Biomedical Information Technology, Shenzhen Institutes of Advanced Technology, Chinese Academy of Sciences, Shenzhen, China; ^5^School of Automation, Nanjing University of Science and Technology, Nanjing, China; ^6^Center for Biosensors, Bioelectronics and Biodevices, University of Bath, Bath, United Kingdom; ^7^Department of Informatics, Modeling, Electronics and Systems, University of Calabria, Rende, Italy; ^8^School of Artificial Intelligence and Automation, Huazhong University of Science and Technology, Wuhan, China

**Keywords:** multimodal data fusion, biomedical signals, data mining and knowledge discovery, deep learning applications, neuroscience

In recent years, sensor and information technologies have greatly boosted the wearable/portal/medical devices development. A large number of multimodal biomedical signals such as electroencephalography (EEG), electrocardiography (ECG), electrooculogram (EOG), and electromyography (EMG), have been recorded for rehabilitation analysis, mental disorders evaluation, emotion recognition, cardiovascular disease diagnosis, etc. In these research fields, most researchers often use single-modal biomedical signals to build the corresponding analysis models. However, many clinical practice tasks, such as disease diagnosis, arrhythmias detection, and sleep condition monitoring, require multimodal biomedical signals together to make correct diagnoses, decisions, identifications, and predictions. It is noted that learning from multimodal biomedical signals can offer the possibility of capturing corresponded information and gaining an in-depth understanding of the relationship among different modalities.

The aim of this topic is to present recent research works to advance the fundamental theory and technologies in biomedical signal processing methods, multimodal fusion algorithms, and biomedical signal-based clinical applications. The special section began with several original researches about the applications of the biomedical signals in specified diseases such as stroke, dysphonia, and visual-spatial neglect (VSN). Three papers explored the applications of EMG signals. Sheng et al. explored the relevance between the increased muscle co-contraction and the corticospinal tract (CST) function in stroke survivors via EMG signals. It demonstrated that the CST and peripheral muscle co-contraction were closely related in stroke survivors. And increasing the intervention of the CST excitability would facilitate the recovery of muscle coordination in the upper limb after stroke. Zhu et al. have measured the speech and the high-density surface EMG signals of the subjects, which suggested that the muscle contraction patterns would be used as a reference of pitch-related phonation functions evaluation. It was a potential alternative method to improve the clinical method for evaluating muscle functions of dysphonia diagnoses, facial paralysis, and other neuromuscular-inclined diseases. Asogbon et al. used KNN, LDA, and RF algorithms to study the impact of various EMG-signal recording duration (SRD) on the characterization of motor intents related with multiple kinds of finger gestures. It demonstrated that choosing a optimal signal length was crucial to characterize multiple classes of targeted limb motions.

The applications of EEG signals in stroke have been explored in three papers. Liang et al. were the first who considered the EEG and functional near-infrared spectroscopy (fNIRS) features as the biomarkers for stroke assessment. The authors have established a linear regression model to predict Berg Balance Scale (BBS) values and used an eightfold cross validation to test the model. It got a result that the EEG features including stroke-related desynchronization (ERD), oxygenated hemoglobin (HBO), and the age were the promising biomarkers for stroke motor recovery. Two researches explored a most common cognitive impairment named visual-spatial neglect (VSN) of poststroke patients. Cao et al. explored the recovery neural substrates of VSN. The study had demonstrated that the dorsal attention networks played a more significant role in recovery from VSN instead of ventral attention networks and the cerebellum was also involved in recovery. Zhang et al. explored the resting-state EEG (rsEEG) features in stroke patients with VSN, which suggested that the resting-state DARAH could differentiate the patient with VSN or not. They demonstrated the resting-state EEG signals would be a useful tool for VSN stroke patients' monitoring and DAR and pdBSI alpha parameters in resting-state EEG could be useful biomarkers.

Aside from EEG and EMG signals, ECG signals are also critical indicators of disease detection. Existing measuring methods of ECG signals don't meet the demands of dynamic measurement. Wang et al. have developed a wearable biosensors system for dynamic ECG monitoring which used a flexible electrode. This system was able to collect high quality ECG signals when subjects exercised. It showed that the proposed electrode could be a potential tool used in long time detection for physiological signal measurements for patients and athletes.

To overcome the effect of physiological signals noises and the single signals' low accuracy, Fu et al. proposed a substructure-based joint probability domain adaptation algorithm (SSJPDA) with bi-projection matrix (BPM) algorithm. The authors used these algorithms to recognize the emotion of subjects based on multimodal fusion physiological data. Compared with other algorithms, the proposed SSJPDA and SSJPDA-BPM algorithms could better deal with noises in data and had improved the performance of emotion recognition.

Deep learning algorithms based on physiological signals were explored in four papers. Li et al. have constructed a centralized steady-state visually evoked potential collaborative brain computer interface (SSVEP-cBCI) system which studied the multi- person EEG features. The system used a transfer learning-based convolutional neural network and three feature fusion methods, which showed the multi-person fusion features achieve more competitive results than single person's. Hu et al. have proposed a generative adversarial network E2SGAN based on EEG-to-stereoelectroencephalography (SEEG), which was aim to synthesize SEEG data from the simultaneous EEG data. E2SGAN is superior to the baseline methods on the real-patient experiments, which demonstrated that the synthesized results had the potential to capture abnormal discharges of the epileptic patients before seizures.

The algorithms about diagnosing the sleep disorders were explored in the other two papers. Chen et al. have creatively proposed a novel method named CNN-BiGRU which consists of considerable spatio-temporal blocks. It was used to classify the sleep apnea (SA) events based on ECG signals. Compared with the state-of-art ECG-based detection methods, CNN-BiGRU demonstrated an obviously competitive result, which could provide sleep monitoring service for the SA detection. Yubo et al. have explored a multimodal attention network MMASleepNet for sleep staging, which can extract the effective features from the multimodal electrophysiological information. Compared with the baseline methods, MMASleepNet performs better in the accuracy and the training speed aspects. It provides a good solution for multimodal sleep monitoring.

## Conclusion and further considerations

To sum up, the papers accepted by this Research Topic mainly explored the biomedical signals applications or novel algorithms based on the detection of diseases in neural science. It enriches the present research studies, some of papers proposed novel methods that achieved better results than baselines. By issuing this Research Topic, it greatly boosts the advancement of multimodal fusion technologies for neuroscience applications based on biomedical signals.

## Author contributions

XF conceptualized this editorial and MQ wrote it. XF, WG, YC, and WM revised this editorial and others significantly contributed to it.

